# Confirmatory Factor Analysis of the Role Performance Scale for Patient Safety Coordinators: Secondary Data Analysis

**DOI:** 10.1002/nop2.70563

**Published:** 2026-04-27

**Authors:** Kyo‐Yeon Park, Kyoungrim Kang

**Affiliations:** ^1^ College of Nursing Pusan National University Yangsan‐Si Republic of Korea; ^2^ College of Nursing, Research Institute of Nursing Science Pusan National University Yangsan‐Si Republic of Korea

**Keywords:** confirmatory factor analysis, patient safety coordinator, role performance, scale

## Abstract

**Aim:**

To develop and validate a scale for predicting and evaluating the role of patient safety coordinators by assessing construct validity and reliability using confirmatory factor analysis.

**Design:**

A methodological study utilising secondary data analysis.

**Methods:**

This methodological study used secondary data from online surveys of 132 patient safety coordinators in small‐ and medium‐sized hospitals. The 25‐item Role Performance Scale, covering four subscales, was validated through confirmatory factor analysis to assess structural validity. Reliability, convergent validity, and discriminant validity were also examined.

**Results:**

The developed 25‐item Role Performance Scale comprised four subscales: Information collection and management, education‐related tasks, patient safety improvement activities, and indicator management. The scale demonstrated strong reliability and validity, with construct reliability values exceeding 0.7 and convergent validity confirmed. Discriminant validity was established as the variance extracted values surpassed inter‐factor correlations. The overall reliability of the scale was high, confirming internal consistency.

**Conclusion:**

The developed scale for patient safety coordinators demonstrated strong validity and reliability, offering an objective assessment method for evaluating key patient safety coordinator tasks and strengthening professional competencies to enhance patient safety systems.

**Patient or Public Contribution:**

No Patient or Public Contribution.

## 1 Introduction

Patient safety is an essential component of quality healthcare, involving the minimisation of unnecessary harm and risk to patients (Institute of Medicine 2001; World Health Organization 2009). Protecting patients from harm is a key responsibility of healthcare organisations and systems (Organization for Economic Co‐operation and Development 2018). The World Health Organization (WHO) designates 17th September each year as World Patient Safety Day to raise international awareness and improve patient safety, thus calling for a systematic approach to promote a safety culture, prevent incidents, and enhance systems (WHO 2018). The Global Patient Safety Action Plan 2021–2030 supports countries in building safer health systems through policy and structural improvements (WHO 2021). Similarly, the Organization for Economic Co‐operation and Development recommends data‐driven monitoring and reporting to strengthen patient safety management systems (Slawomirski et al. 2017), thus emphasising the significance of systemic change and patient–provider collaboration.

Korea promulgated the Patient Safety Act in January 2015 and fully implemented it in July 2016 in response to growing social concerns about patient safety (Kim 2016). The Act aimed to create a legal and institutional foundation for preventing patient accidents and ensuring a safe environment for the public (Korea Institute for Healthcare Accreditation 2020). It requires medical institutions above a certain size to appoint a dedicated patient safety coordinator and strengthen standards for establishing patient safety committees to implement a patient safety management system (Korea Law Information Center 2020). These changes have strengthened Korea's healthcare system, further emphasising the importance of dedicated patient safety coordinators (Korea Law Information Center 2020).

In other countries, patient safety officers, specialists, and similar personnel are crucial in improving patient safety by managing incident reporting systems, implementing prevention policies, and educating medical staff (Kwak et al. 2020). In Korea, dedicated patient safety coordinators are those who work exclusively on patient safety and medical quality improvement in medical institutions over a certain size (Korea Law Information Center 2020), and are differentiated by the fact that their deployment is mandatory based on legal grounds. As 87.2% of annual patient safety incidents are reported through patient safety coordinators (Korea Institute for Healthcare Accreditation 2018), patient safety coordinators actively report and share information on patient safety incidents (Ministry of Health and Welfare 2023). They also lead investigations, conduct analyses, and oversee practical work and education to improve medical care quality (Park and Kang 2022).

In Korea, the 2nd National Patient Safety Plan (2023–2027) has identified the establishment of a safety management system as a key priority to enhance the patient safety capabilities of healthcare institutions (Ministry of Health and Welfare 2023). In particular, the mandatory deployment of dedicated patient safety coordinators has been expanded to small‐ and medium‐sized healthcare institutions to strengthen the patient safety infrastructure. Accordingly, it is essential for these coordinators to effectively perform their roles and enhance their competencies (Ministry of Health and Welfare 2023).

Role performance refers to the extent to which individuals effectively carry out the behaviours expected of them within an organisation (Katz and Kahn 1978). In the healthcare setting, role clarity, task execution, and interdisciplinary collaboration are regarded as core elements of effective role performance (Manser 2009; Lingard et al. 2004). The WHO has emphasised that standardised protocols and clearly defined roles are directly linked to improved safety outcomes across various healthcare systems (Leotsakos et al. 2014). Similarly, the Canadian Patient Safety Institute (2020) (CPSI) identifies communication, teamwork, and risk management competencies as essential components of the patient safety coordinator's role (CPSI 2020). These international perspectives highlight that clearly delineated professional roles and continuous competency development are critical for improving safety culture and reducing preventable harm in healthcare environments (Abikenova et al. 2023; Reeves et al. 2017).

International patient safety frameworks further emphasise the importance of clearly defined competencies and role performance in promoting safe healthcare systems (WHO 2021). The WHO and the CPSI have identified core competencies for patient safety professionals, including incident reporting, risk management, safety education, and system improvement activities (WHO 2021; CPSI 2020). These competencies highlight the need for healthcare professionals responsible for patient safety to collect and manage safety information, facilitate organisational learning, implement improvement strategies, and monitor safety indicators (CPSI 2020). In addition, recent research on nursing role performance and patient safety competencies has demonstrated that effective safety leadership requires not only technical knowledge but also the ability to coordinate safety programs, educate healthcare staff, and support organisational safety culture (Abikenova et al. 2023; Reeves et al. 2017). The study on the nursing workforce has also emphasised that structured role definitions and validated measurement tools are essential for evaluating professional performance and strengthening patient safety systems (Abikenova et al. 2023). These international frameworks suggest that the role of patient safety coordinators involves multidimensional competencies related to safety information management, education, improvement activities, and performance monitoring (CPSI 2020; WHO 2021).

However, patient safety coordinators in Korea face challenges in securing expertise due to overwhelming workload and the lack of clear guidelines regarding task scope and responsibilities (Park et al. 2020; Korea Institute for Healthcare Accreditation 2018). In contrast, individuals in similar positions in other countries perform specialised roles with systematic support, thereby contributing to more robust patient safety systems (Kwak et al. 2020). To strengthen the effectiveness of patient safety coordinators in Korea, it is essential to clearly define their roles, establish a structured scope of responsibilities, and enhance their professional competencies. According to Nieva and Sorra (2003), fostering a culture of safety requires the active engagement of designated personnel who promote shared values, mutual trust, and open communication throughout theorganisation. In this context, patient safety coordinators play a pivotal role in facilitating cultural change and reinforcing safety‐related behaviours across all levels of the healthcare system.

In Korea, the Patient Safety Act Operational Manual defines the primary responsibilities of dedicated patient safety coordinators in four areas (Korea Institute for Healthcare Accreditation 2020): Information collection and management, education‐related tasks, patient safety improvement activities, and indicator management. Based on these key work elements, a 25‐item Role Performance Scale was developed by Park and Kang (2022). However, the scale's construct validity was assessed only through exploratory factor analysis (EFA), and confirmatory factor analysis (CFA), which is essential to test the hypothesised structure, was not performed. Therefore, this study conducted a CFA based on 25 preliminary items derived from Park and Kang (2022) to verify the construct validity and reliability of the scale to refine it into a measure that can predict and evaluate the role performance of patient safety coordinators. The study enhanced the reliability and usability of the existing scale, thus providing basic data to strengthen the expertise of patient safety coordinators and improve the efficiency of their roles.

## 2 Materials and Methods

### 2.1 Study Design

This study is a secondary data analysis that uses research data from Park and Kang (2022) on patient safety coordinators working in small‐ and medium‐sized hospitals. It is a methodological study that conducts a CFA of the role performance scale developed for patient safety coordinators.

### 2.2 Participants and Data Collection

Data collection was conducted through an online questionnaire over a 3‐week period, from 8th February to 27th February 2022. Participants provided consent after being informed about the study's purpose, methods, participation criteria, survey response process, data confidentiality, and voluntary participation.

The participants of the original study were dedicated patient safety coordinators working in small‐ and medium‐sized hospitals, selected based on the criteria of being registered with the Ministry of Health and Welfare as coordinators in general hospitals or hospitals with 100–300 beds. Exclusion criteria included those on leave and those who had been in the role for < 1 month owing to the adaptation period. In this study, we recruited 132 participants from the original data and excluded 11 patient safety coordinators working in hospitals with over 300 beds, leaving 121 participants for the analysis. However, all 132 participants, including those excluded, were included in the secondary data analysis. According to the recommended sample size of 5–10 times the number of questions (Hair et al. 2019), the sample size was considered adequate for this study.

Raw data were collected with the approval of the Institutional Review Board (IRB) of Pusan National University (PNU IRB/2021_183_HR). No personally identifiable information was collected, and the data were computer‐coded. The anonymised raw data were securely stored on the co‐researcher's personal laptop and were only accessible to the principal investigator and co‐researcher. This study was approved by the Institutional Review Board (IRB) of Pusan National University (PNU IRB/2024_06_HR).

### 2.3 Instruments

#### 2.3.1 General and Work‐Related Characteristics

Participant characteristics included general factors (age, sex, marital status, education, occupation, salary, and motivation to choose) and work‐related factors (type of medical institution, number of beds, department, adjunct status, experience in evaluating medical institutions, clinical experience, and work experience of dedicated staff) from the original data.

#### 2.3.2 Role Performance

The role performance scale developed by Park and Kang (2022) was confirmed for reliability and validity through EFA and content validity verification based on the main task elements outlined in the Patient Safety Act Operational Manual (Korea Institute for Healthcare Accreditation 2020). The scale comprises 25 items, divided into four subscales: Information collection and management, education‐related tasks, patient safety improvement activities, and indicator management. It uses a 5‐point Likert scale ranging from 1 (‘never’) to 5 (‘always’), with higher scores indicating significant role performance. The reliability at the time of development was Cronbach's α = 0.94.

### 2.4 Data Analysis

To verify the reliability and validity of the developed scale, data were analysed using SPSS/WIN 27.0 and AMOS/WIN 26.0. Prior to analysis, the dataset was screened for missing values, and no missing data were identified. Descriptive statistics, including frequencies, percentages, means, and standard deviations, were used to analyse the general and work‐related characteristics of the participants. CFA was employed to verify construct validity.

First, the Kaiser–Meyer–Olkin (KMO) test and Bartlett's test of sphericity were performed to assess the suitability of the data for factor analysis. A KMO value of ≥ 0.6 is considered suitable for factor analysis, and *p* < 0.05 for Bartlett's test indicates the significance of the correlation matrix. Factors were extracted using principal component analysis with Varimax rotation, with an eigenvalue ≥ 1.0 as the criterion for extraction. Factor loading (FL) was used as an item selection criterion, with a minimum recommended value of ≥ 0.30 (Hair et al. 2019). CFA was performed by anchoring the items in each subscale based on the existing Patient Safety Act Operations Manual (Korea Institute for Healthcare Accreditation 2020).

Maximum likelihood estimation was used to estimate the model, and the assumption of normality of the data was verified. Prior to conducting the analyses, the normality of the data was assessed by examining skewness and kurtosis values. All items showed skewness values below |3| and kurtosis values below |10|, indicating that the assumption of normality was satisfied (Hair et al. 2019). Multicollinearity was assessed by examining inter‐factor correlations. All correlation coefficients were below00.85, indicating that multicollinearity was not a concern. Model fit was assessed using absolute and incremental fit indices, including the root mean square error of approximation (RMSEA), comparative fit index (CFI), and goodness‐of‐fit index (GFI). To improve model fit, covariances were added for items with a modification index (MI) > 10.0 within the same latent factor (Byrne 2016). The addition of residual covariances was theoretically justified based on conceptual overlap and similarity in wording among items within the same factor (Brown 2015). Average variance extracted (AVE) and construct reliability (CR) were calculated to verify the convergent and discriminant validity of the items. Convergent validity was achieved when the AVE exceeded 0.5, and the CR exceeded 0.7. Discriminant validity was assessed by determining whether the AVE value surpassed the square value of the inter‐factor correlation coefficient. To assess overall scale and factor reliability, internal consistency was evaluated using Cronbach's α. Values of 0.9 or higher were considered excellent, 0.7–0.9 good, 0.6–0.7 acceptable, and less than 0.6 unacceptable.

## 3 Results

### 3.1 General and Work‐Related Characteristics of the Participants

The general and work‐related characteristics of the participants are presented in Table 1. The mean age of the participants was 40.51 years, and 97.0% (*n* = 128) were female. Most participants were nurses (99.2%, *n* = 131), and a bachelor's degree was the most common level of education (60.6%, *n* = 80). Regarding selection motivation, 40.9% (*n* = 54) of participants assumed the role of patient safety coordinator due to hospital or supervisor instructions (involuntary), while 37.9% (*n* = 50) chose the role based on factors such as interest in patient safety or preference for the type of work (voluntary). This variable was included to provide contextual understanding of how individuals entered the patient safety coordinator role. With respect to hospital characteristics, 36.4% (*n* = 48) of participants worked in general hospitals, and 41.7% (*n* = 55) worked in hospitals with fewer than 250–300 beds. In terms of departmental affiliation, 50.8% (*n* = 67) belonged to nursing departments (part‐time), 22.0% (*n* = 29) to administration (part‐time), and 26.5% (*n* = 35) to other departments. Additionally, 62.1% (*n* = 82) of participants performed patient safety duties alongside other responsibilities, and 78.0% (*n* = 103) were involved in healthcare organisation accreditation assessments. The average total work experience was 14.32 years, and the mean duration of working as a patient safety coordinator was 2.41 years.

**TABLE 1:** General and work‐related characteristics of the participants (*n* = 132).

Variables	Categories	*n* (%) or m ± SD		
General characteristics				
Age (year)				40.51 ± 8.13
Gender	Female	128	97.0	
	Male	4	3.0	
Occupation	Nurse	131	99.2	
	Doctor	1	0.8	
Education level	Bachelor's degree	31	23.5	
	RN‐BSN	80	60.6	
	Master's degree or higher	21	15.9	
Selection motivation	Type of work	50	37.9	
	Hospital or supervisor instructions	54	40.9	
	Interest in patient safety	27	21.2	
Work‐related Characteristics				
Type of medical institution	Convalescent hospital	48	36.4	
	General hospital	48	36.4	
	Hospital	36	27.2	
Number of beds	More than 100 to less than 200 beds	23	17.4	257.55 ± 105.39
	More than 200 to less than 250 beds	43	32.6	
	More than 250 to less than 300 beds	55	41.7	
	300 beds or more	11	8.3	
Affiliation department	Solo	35	26.5	
	Nursing (part)	67	50.8	
	Administrative (part)	29	22.0	
	Medical Service (part)	1	0.7	
Concurrent position	Performed with other duties	82	62.1	
	Exclusively patient safety	50	37.9	
Medical institution certification	Enforced	103	78.0	
	Not enforced	29	22.0	
Total work experience (years)	Less than 10	41	31.0	14.32 ± 8.18
	More than 10 to less than 15	29	22.0	
	More than 15 to less than 20	33	25.0	
	20 or more	29	22.0	
Experience as a dedicated patient safety coordinator (years)	Less than 1	26	19.7	2.41 ± 2.03
	More than 1 to less than 3	60	45.5	
	More than 3 to less than 5	33	25.0	
	5 years or more	13	9.8	

*Note:* This table presents the demographic and work‐related characteristics of the patient safety coordinators, including age, gender, marital status, education level, occupation, annual salary, selection motivation, type of medical institution, number of beds, affiliation department, concurrent position, medical institution certification, total work experience, and experience as a dedicated patient safety coordinator.

### 3.2 Verification

#### 3.2.1 Item and Factor Analysis

KMO and Bartlett's tests of sphericity were conducted to determine whether the 25 items were suitable for factor analysis. The KMO result was 0.91, and Bartlett's test of sphericity showed a statistically significant difference (χ2 = 2263.36, *p* < 0.001), indicating that the items were suitable for factor analysis.

To maintain the independent relationship between the items and identify the characteristics of the factors, a principal component analysis using varimax rotation was conducted, and four factors with eigenvalues > 1.0 were extracted. Additionally, the minimum criterion for FLs was 0.30 (Hair et al. 2019); all 25 items were significantly loaded with FLs ≥ 0.30 and were entered into the measurement model of CFA.

#### 3.2.2 Confirmatory Factor Analysis

**FIGURE 1:**
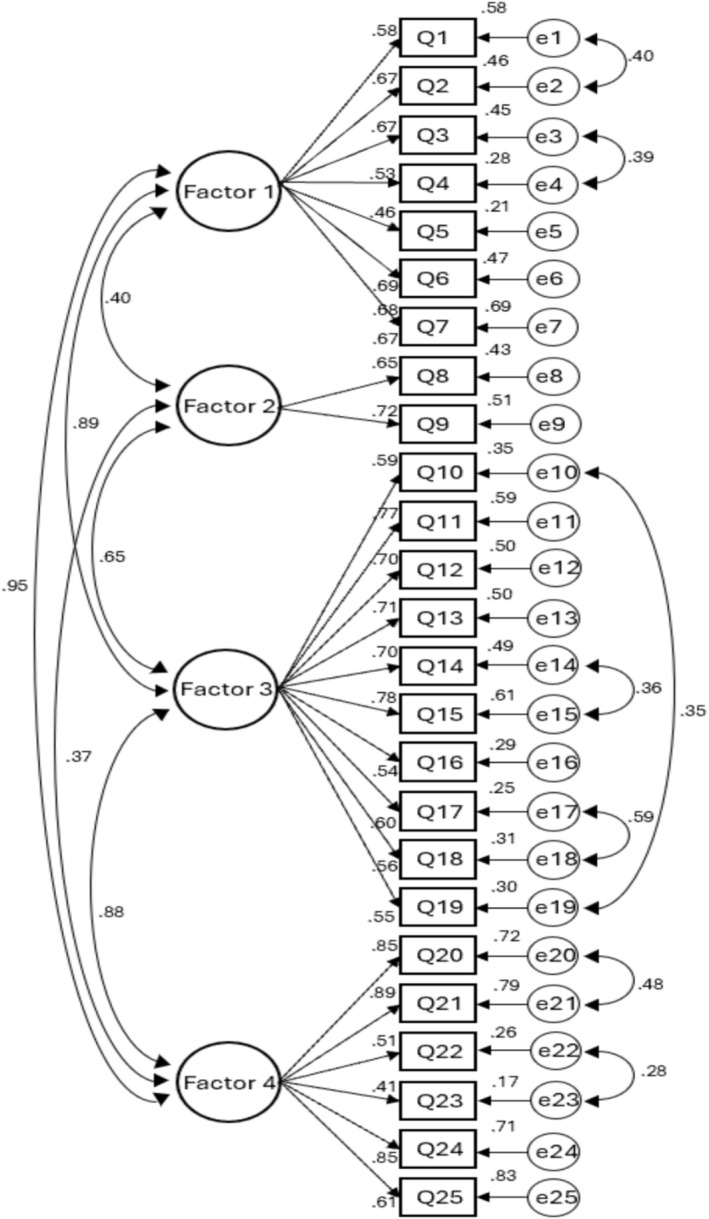
Confirmatory factor analysis of the alternative model. This figure illustrates the structural relationships among the four latent factors—information collection and management, education, patient safety improvement activities, and indicator management—demonstrating the validity and reliability of the proposed measurement model.

A CFA was conducted by constructing a measurement model representing the relationship between the latent factors (subscales of the main work factors) and the observed variables (measurement items) based on the existing operating manual of the Patient Safety Act Operational Manual (Korea Institute for Healthcare Accreditation 2020). This model was subsequently estimated using the maximum likelihood method. The fit of the model was evaluated using RMSEA (an absolute fit index), GFI, and CFI (both incremental fit indices). For RMSEA, values between 0.05 and 0.10 are considered acceptable, while GFI and CFI values of at least 0.70 and 0.90, respectively, indicate an acceptable model fit (Hair et al. 2019). The initial analysis showed χ2 = 576.400 (df = 269), *p* < 0.001, RMSEA = 0.112, GFI = 0.691, and CFI = 0.795, indicating that the model fit was not acceptable. Although item deletion was considered as a potential approach to improve model fit, all items were retained because they were developed based on the roles specified in the Patient Safety Act Operational Manual (Korea Institute for Healthcare Accreditation 2020) and their content validity had been confirmed through expert review. MI was determined to improve the model fit. MI is used to reduce the χ2 value by establishing correlations between errors within the same latent factor, and correlations were added to items with more than 10 items in the same latent factor. As adding correlations in the process of covariance correction affects the overall model fit, this study repeatedly conducted CFA by adding correlations one at a time, and the MI within the same latent factor was found to be between 10.220 and 37.924. These item pairs addressed closely related operational tasks, which may have resulted in correlated measurement errors. Moreover, this study sequentially added covariances to the errors of Items 1, 2, 3, and 4 of information collection and management; Items 10 and 19 of patient safety improvement activities; Items 14 and 15; Items 17 and 18; Items 20 and 21 of indicator management; and Items 22 and 23 of patient safety improvement activities (Figure 1). The final analysis resulted in χ2 = 507.124 (df = 262), *p* < 0.001, RMSEA = 0.096, GFI = 0.736, and CFI = 0.853, indicating that the model fit of the measurement model of the relationship between the four latent factors and the observed variables was adequate. To further evaluate the structural validity of the scale, an alternative CFA model assuming a single‐factor structure was also tested. The one‐factor model showed poorer model fit (χ^2^ = 1120.342, df = 275, *p* < 0.001, RMSEA = 0.165, CFI = 0.502, GFI = 0.412) compared with the four‐factor model, supporting the multidimensional structure of the scale (Table 2).

**TABLE 2:** Model fit indices for the confirmatory factor analysis (*n* = 132).

	χ2	df	*p*	RMSEA	GFI	CFI
One‐factor	1120.342	275	< 0.001	0.165	0.502	0.412
Initial	576.400	269	< 0.001	0.112	0.691	0.795
Modified	507.124	262	< 0.001	0.096	0.736	0.853

*Note:* The table provides the results of the initial and modified models, including chi‐square (χ^2^), degrees of freedom (df), *p*‐value, root mean square error of approximation (RMSEA), goodness‐of‐fit index (GFI), and comparative fit index (CFI), demonstrating the improvement in model fit after modification.

Abbreviations: CFI, comparative fit index; GFI, goodness‐of‐fit index; RMSEA, root mean square error of approximation.

#### 3.2.3 Convergent and Discriminant Validity of Questions

To verify the convergent and discriminant validity of the items through CFA, the AVE and CR were assessed (Table 3). The AVE values ranged from 0.502 to 0.576, all exceeding the recommended threshold of 0.50, and the CR values ranged from 0.705 to 0.908, all above the standard of 0.70, thus confirming the convergent validity of the items (Polit and Beck 2008). Discriminant validity was assessed using the correlation coefficient (ρ) and standard error (SE). The range of SE, multiplied by two and added or subtracted from the correlation coefficient, was between 0.708 and 0.759, which did not include the cut‐off value of 1, thus confirming discriminant validity (Polit and Beck 2008).

**TABLE 3:** Convergent validity of the model (*n* = 132).

Item		Standard estimates	SE	AVE	CR
Information Collection and Management				0.506	0.874
1	An internal reporting system for patient safety incidents is established.	0.762	0.240		
2	Incident/accident reports are submitted within the designated timeframe following the occurrence of patient safety incidents.	0.675	0.477		
3	Root Cause Analysis (RCA) is conducted for patient safety incidents that occur in the hospital.	0.668	0.580		
4	Failure Mode and Effects Analysis (FMEA) is performed at least once a year to prevent patient safety incidents.	0.528	0.507		
5	Incident reports are submitted in accordance with the ‘Korea Patient Safety Reporting & Learning System.’	0.460	0.686		
6	Statistics on reported patient safety incidents are managed by the hospital.	0.687	0.323		
7	The results of patient safety incident analyses are shared with staff and management.	0.833	0.248		
Education				0.545	0.705
8	Education programs for healthcare professionals on patient safety are actively conducted.	0.654	0.412		
9	Education programs for patients and their caregivers on patient safety are actively conducted.	0.717	0.374		
Patient Safety Improvement Activities				0.502	0.908
10	Improvement activities are actively carried out following the occurrence of patient safety incidents.	0.588	0.382		
11	The results of patient safety improvement activities are shared with staff and management.	0.767	0.277		
12	The Patient Safety Committee is operated with the inclusion of the management team.	0.704	0.441		
13	Detailed guidelines for patient safety standards are in place.	0.705	0.353		
14	Compliance with patient safety standards by relevant departments or staff is monitored.	0.697	0.345		
15	Compliance with patient safety standards and areas for improvement are shared with staff and management.	0.780	0.345		
16	As a patient safety coordinator, you receive training that is practically beneficial for duties specific to your hospital beyond mandatory education.	0.542	0.521		
17	Patient safety culture is periodically evaluated to promote its improvement.	0.500	0.502		
18	Activities to improve patient safety culture are implemented.	0.555	0.508		
19	Medical staff and management actively participate in fostering and enhancing a patient safety culture within the hospital.	0.546	0.468		
Indicator Management				0.576	0.883
20	A system for managing patient safety indicators is established.	0.850	0.208		
21	Patient safety indicators are selected and implemented.	0.888	0.163		
22	Data related to the hospital's patient safety is provided to institutions designated by Presidential Decree, such as the Central Patient Safety Centre, for purposes such as developing patient safety indicators.	0.506	0.941		
23	Training for personnel responsible for each patient safety indicator is conducted.	0.406	0.928		
24	The results of patient safety indicators are produced.	0.845	0.175		
25	The hospital shares the results of patient safety indicators with staff, medical personnel, and management.	0.913	0.148		

*Note:* This table reports the standardised estimates, standard errors (SE), average variance extracted (AVE), and construct reliability (CR) values for each of the four subscales: Information collection and management, education, patient safety improvement activities, and indicator management, confirming the convergent validity of the scale.

### 3.3 Reliability Test

To assess the reliability of the patient safety coordinator role performance scale, the internal consistency reliability coefficient, Cronbach's α, was calculated for the entire 25‐item scale. The Cronbach's α was 0.941 for the overall scale, 0.859 for information collection and management, 0.889 for patient safety improvement activities, and 0.865 for indicator management.

The education‐related tasks subscale, which consisted of only two items, showed a relatively lower internal consistency (Cronbach's α = 0.637), which is acceptable but limited. Given the small number of items, this subscale was further evaluated using the Spearman–Brown coefficient, in accordance with the recommendation of Hoeppner et al. (2011) that two‐item scales should be assessed using alternative reliability indicators. The resulting Spearman–Brown coefficient was 0.643, indicating an acceptable level of reliability for a two‐item subscale.

In addition, to complement the evaluation of internal consistency reliability, a split‐half reliability analysis was conducted. The items were divided into two parts based on odd and even numbers, and the Spearman–Brown corrected coefficient was calculated. The result was 0.940, indicating that the scale demonstrates a high level of measurement consistency and reliability.

## 4 Discussion

This study was conducted to verify the reliability and validity of a measurement tool developed by Park and Kang (2022), which was based on key task elements outlined in the Patient Safety Act Operational Manual (Korea Institute for Healthcare Accreditation 2020), to objectively assess the role performance of patient safety coordinators. The original scale developed by Park and Kang (2022) was validated only through EFA, without confirming the interrelationships among the factors. Therefore, in this study, CFA was conducted to verify the structural validity of the original scale, and EFA was also performed to examine the FLs and select appropriate items. The final CFA model in this study was structured around four factors corresponding to the main work elements outlined in the Patient Safety Act Operational Manual (Korea Institute for Healthcare Accreditation 2020).

The structure of this scale was developed based on nationally standardised guidelines to ensure consistency in defining the roles and responsibilities of patient safety coordinators; however, it was based primarily on the Patient Safety Act Operational Manual, which served as the main reference for patient safety activities in Korea and was the most practical and widely accepted standard at the time of the scale's development (Korea Institute for Healthcare Accreditation 2020). However, relying solely on this manual may weaken the theoretical validity of the scale and limit its applicability in diverse healthcare settings. To address this limitation, future research should consider incorporating internationally established theories and role frameworks in patient safety to broaden the conceptual foundation of the scale and improve its applicability across various healthcare contexts.

CFA uses various fit indices to evaluate how well the hypothesised model aligns with the observed data (Hu and Bentler 1999; Byrne 2016). CFA was evaluated using absolute fit indices (RMSEA) and incremental fit indices (GFI and CFI) in this study. RMSEA assesses how well the model fits the data (Hair et al. 2019). In this study, the RMSEA value was 0.096, which falls within the acceptable range of 0.05 to 0.10, indicating that the model sufficiently explains the data. GFI measures how well the observed data are explained by the model (Hair et al. 2019). The GFI value of 0.736 in this study falls below the threshold of 0.70 but still suggests that the model explains the data to some extent. CFI evaluates the relative fit of the model compared to a baseline model (Hair et al. 2019). The CFI value of 0.853 in this study exceeds the minimum threshold of 0.70, supporting the adequacy of the model's relative explanatory power. These results demonstrate that the scale, consisting of four factors—information collection and management, education, patient safety improvement activities, and indicator management—effectively explains the observed data. Therefore, this scale is appropriate for assessing the role performance of dedicated patient safety coordinators by reflecting both theoretical structure and practical characteristics.

The convergent validity of the items was verified through the AVE and CR values, and all subfactors met the validation criteria, confirming that each factor accurately measured the converging correlations. The AVE value was greater than 0.5, and the CR value was greater than 0.7, indicating that the items in each factor effectively described the same factor. The discriminant validity test showed that the AVE value was greater than the squared value of the inter‐factor correlation coefficient, which met the validation criteria, indicating that each factor measured the concepts independently and without overlap. Therefore, the seven items on information collection and management, two items on education‐related tasks, ten items on patient safety improvement activities, and six items on indicator management sufficiently described each factor and were distinct from the items included in the other factors. These results suggest that it is feasible to calculate scores for each sub‐factor, allowing for a specific and independent assessment of each factor. Therefore, the scale has structural validity in reliably and validly assessing the performance of patient safety coordinators by sub‐factors.

In the initial model of CFA conducted in this study, some fit indices were found to be inadequate. Therefore, while item deletion was considered as a method to improve model fit, all items were retained because they were developed based on the roles specified in the Patient Safety Act Operational Manual (Korea Institute for Healthcare Accreditation 2020) and their content validity was confirmed through expert review. This is because removing items could have resulted in the scale failing to sufficiently capture the legally defined roles of patient safety coordinators. Accordingly, to improve model fit while maintaining theoretical coherence, a total of seven error covariances were added between items within the same latent factor (Byrne 2016). These modifications were applied conservatively—only when the MI exceeded 10 and when theoretical justification was present. According to (Hair et al. 2019), post hoc modifications such as the addition of error covariances are acceptable when grounded in theory and limited to within‐factor relationships. This approach is particularly appropriate in nursing research, where items often share similar terminology, measurement context, or content focus, which may lead to correlated measurement errors (Brown 2015). However, these modifications may have optimised the model to the current sample, and this limitation should be taken into account when applying the scale. Therefore, further validation with diverse samples and settings is needed to enhance the generalisability and validity of the scale.

Reliability of the scale for measuring the role performance of patient safety coordinators was assessed using Cronbach's α to evaluate internal consistency, with the overall reliability showing a Cronbach's α of 0.941, indicating an excellent level of reliability and confirming high internal consistency. Additionally, the scale consists of four subscales: Information collection and management, education‐related tasks, patient safety improvement activities, and indicator management. The reliability of each subscale was assessed as follows: The information collection and management subscale, which includes seven items related to the establishment of a patient safety incident reporting system, incident analysis, statistics, and sharing within healthcareorganisations, demonstrated a Cronbach's α of 0.859, indicating a high level of reliability. The patient safety improvement activities subscale, which includes ten items related to post‐incident improvements, patient safety committee activities, ensuring staff compliance with patient safety standards, and fostering a patient safety culture, demonstrated a Cronbach's α of 0.889. This value indicates good reliability. The indicator management subscale, which consisted of six items related to the establishment and implementation of the patient safety indicator system and managing improvements, demonstrated a Cronbach's α of 0.865, also indicating good reliability. The education‐related tasks subscale, which comprises only two items, showed a Cronbach's α of 0.637. Although this value is somewhat low, it falls within the acceptable range (0.60 ~ 0.70). The relatively low reliability may be attributed to the fact that the subscale consists of only two items and that the intended target audiences of each item differ—patients and caregivers vs. healthcare professionals—which may lead to significant differences in educational needs, content, and delivery methods. As noted by Hoeppner et al. (2011), subscales with only two items tend to show lower Cronbach's α values due to item number limitations, which may underestimate internal consistency. Therefore, in line with Hoeppner et al. (2011)'s recommendation, we additionally calculated the Spearman–Brown coefficient as an alternative reliability indicator, yielding a value of 0.643, indicating an acceptable level of reliability for a two‐item subscale. A supplementary analysis also confirmed that excluding either of the two items had a minimal effect on the overall reliability of the full scale. Given that the education‐related tasks subscale is a legally mandated responsibility of patient safety coordinators in the Korean healthcare system, the subscale was retained to preserve content validity. Although the internal consistency of this subscale was relatively lower than that of the other subscales, it was retained based on both theoretical and practical considerations, particularly its role as a legally mandated responsibility of patient safety coordinators and its importance in preserving the content validity of the scale. Nevertheless, future research should focus on developing additional items that can more fully reflect the educator role of patient safety coordinators, which would help improve internal consistency and more accurately capture this key aspect of their responsibilities.

Each subscale score derived from the scale provides practical insights into specific areas of role performance. A high score in the ‘information collection and management’ subscale suggests that the coordinator is actively engaged in managing incident reporting systems, analysing patient safety data, and sharing information across theorganisation. A high score in the ‘education‐related tasks’ subscale indicates frequent provision of safety education to healthcare staff, patients, or caregivers, reflecting the coordinator's contribution to safety awareness and knowledge dissemination. Similarly, elevated scores in ‘patient safety improvement activities’ imply proactive involvement in post‐event reviews, committee participation, and safety culture initiatives, while high scores in ‘indicator management’ reflect systematic oversight and continuous monitoring of safety performance metrics. By interpreting subscale scores in this way, healthcare institutions can identify both strengths and areas needing support in the Patient Safety Coordinators' role execution, thereby facilitating more targeted policy and organisational interventions. A summary table based on COSMIN criteria was added to clarify which psychometric properties were evaluated in this study and which require further investigation (Table 4).

**TABLE 4:** Summary of psychometric properties of the scale based on COSMIN criteria.

Measurement property	Method used in this study	Results	COSMIN evaluation
Structural validity	CFA	RMSEA = 0.096, CFI = 0.853, GFI = 0.736	Sufficient
Internal consistency	Cronbach's α	Total scale α = 0.941; subscales α = 0.637–0.889	Sufficient
Convergent validity	AVE, CR	AVE = 0.502–0.576; CR = 0.705–0.908	Sufficient
Discriminant validity	Correlation analysis and AVE comparison	AVE values exceeded squared inter‐factor correlations	Sufficient
Test–retest reliability	Not assessed	—	Not tested
Criterion validity	Not assessed	—	Not tested
Measurement invariance	Not assessed	—	Not tested
Responsiveness	Not assessed	—	Not tested

*Note:* This table summarises the psychometric properties of the scale according to the COSMIN (Consensus‐based Standards for the Selection of Health Measurement Instruments) framework. It indicates which measurement properties were evaluated in this study, including structural validity, internal consistency, convergent validity, and discriminant validity, as well as properties that were not assessed, such as test–retest reliability, criterion validity, measurement invariance, and responsiveness.

Abbreviations: AVE, average variance extracted; CFA, confirmatory factor analysis; CFI, comparative fit index; COSMIN, consensus‐based standards for the selection of health measurement instruments; CR, construct reliability; GFI, goodness‐of‐fit index; RMSEA, root mean square error of approximation.

This study has limitations. First, criterion validity was not assessed using standardised measurement scales commonly employed in related fields. In addition, test–retest reliability and measurement invariance across different groups were not examined because the present study used cross‐sectional secondary data. Therefore, future studies should evaluate these additional psychometric properties, including temporal stability and criterion‐related validity using external benchmarks, to further confirm the robustness of the scale. Furthermore, the responsiveness of the scale—its ability to detect changes in role performance over time—was not assessed in this study. Future longitudinal or intervention‐based research is warranted to examine whether the scale can sensitively capture changes in role performance. Another limitation is that the sample consisted exclusively of patient safety coordinators working in small‐ and medium‐sized hospitals in Korea, and the participants were highly homogeneous in terms of gender and profession, as the vast majority were Korean female nurses. Because the scale was developed and validated based on this specific group, it reflects the characteristics of the Korean patient safety system and may not be directly applicable to other countries or healthcare settings. Future research should include more diverse samples across different countries, healthcare systems, and institutional sizes, including larger hospitals and patient safety coordinators from various professional backgrounds beyond nursing, to enhance the external validity and generalisability of the scale. Nevertheless, this study is significant in that it verified the validity and reliability of a scale designed to measure the role performance of dedicated patient safety coordinators in Korea. The scale provides a clear and concrete definition of the scope of tasks and responsibilities for patient safety coordinators and offers a basis for objectively assessing their role performance. This scale is expected to contribute to strengthening patient safety capabilities in healthcare organisations and to facilitating the development of systematic management strategies.

## 5 Conclusion

This study verified the validity and reliability of a scale developed by Park and Kang (2022) based on the main task elements of the Patient Safety Act Operational Manual (Korea Institute for Healthcare Accreditation 2020). The scale consists of 25 items divided into four subscales: Information collection and management, education, patient safety improvement activities, and indicator management. Each item is rated on a 5‐point Likert scale, with higher scores indicating greater role performance.

The scale provides a framework for objectively evaluating the scope and effectiveness of tasks performed by patient safety coordinators. It contributes to enhancing their professionalism and improving patient safety management systems. Future research should further validate the scale in diverse settings and explore its broader applicability.

## Author Contributions

Conceptualisation: Kyo‐Yeon PARK, Kyoungrim KANG; Data curation: Kyo‐Yeon PARK; Formal analysis: Kyo‐Yeon PARK; Writing – original draft: Kyo‐Yeon PARK; Writing – review and editing: Kyo‐Yeon PARK, Kyoungrim KANG; Supervision: Kyoungrim KANG.

## Funding

The authors have nothing to report.

## Ethics Statement

This study was conducted in accordance with the Declaration of Helsinki. Ethical approval for data collection was obtained from the Institutional Review Board of Pusan National University (Approval No. PNU IRB/2021_183_HR). The secondary analysis of this data was conducted after receiving an exemption from IRB, as no new data were collected and only de‐identified datasets were used (Exemption No. PNU IRB/2024_06_HR). All personally identifiable information was removed before data access, and the dataset was securely stored and analysed.

## Consent

The primary study obtained written informed consent from all participants before data collection. In the present study, only de‐identified secondary data, provided by the original researchers, wereanalysed. No personally identifiable information was accessed or used. Given that this study involved secondary data analysis without direct participant interaction, the requirement for additional informed consent was waived by the Institutional Review Board of Pusan National University (Exemption No. PNU IRB/2024_06_HR).

## Conflicts of Interest

The authors declare no conflicts of interest.

## Data Availability

The data that support the findings of this study are available from the corresponding author upon reasonable request.
